# To Use or Not Use Car Sharing Mobility in the Ongoing COVID-19 Pandemic? Identifying Sharing Mobility Behaviour in Times of Crisis

**DOI:** 10.3390/ijerph19053127

**Published:** 2022-03-07

**Authors:** Maria del Mar Alonso-Almeida

**Affiliations:** Department of Business Organization, Faculty of Economics and Business Administration, Universidad Autónoma de Madrid, Ciudad Universitaria de Cantoblanco, 28049 Madrid, Spain; mar.alonso@uam.es

**Keywords:** car sharing usage, pandemic times, sharing mobility, shared mobility

## Abstract

Car sharing services have expanded in order to meet the new necessities of mobility worldwide in an innovative way. Before the COVID-19 pandemic, car sharing was a very popular mode of transportation among young adults in big cities. However, during this ongoing pandemic and with public transportation considered a super-spreading transmitter, the usage of car sharing is unclear. Therefore, the aim of this study, which is explorative in nature, is to investigate the usage, advantages, drivers, and barriers to car sharing during this ongoing pandemic era. To this end, 66 interviews were conducted among users of car sharing during the COVID-19 pandemic. The findings provide key information for the planning of car sharing operations and public transportation in the context of avoiding COVID-19 infection and respecting the recommendations of local governments. In addition, new emerging profiles of car sharing users in the ongoing pandemic are identified. This research provides relevant insights for both business practice and policy makers.

## 1. Introduction

The COVID-19 pandemic has spread rapidly worldwide, becoming a major threat to global public health [1]. Mobility has been severely affected due to lockdowns and the different waves of the pandemic. Governments have legislated mobility restrictions and advised about the danger of infections on public and shared modes of transportation due to the fact that they are small enclosed spaces shared by a high number of people. 

During past pandemics, travelling was perceived as one of the main channels of transmission and a source of infections [2]. In fact, the perception of possible COVID-19 infection in different modes of public transportation such as buses, trains or aeroplanes [3] has been exacerbated due to several factors. First, viral transmission rates seem to increase in small and overcrowded spaces. Second, studies (for example, [3]) predicted that approximately 25% of COVID-19 infections could occur during the use of public transport. Third, research indicated that the virus was stable on surfaces and different materials for days [4]. Finally, research (for example, [3]) confirmed a significant positive relationship between use of public transport and the number of COVID-19 cases/deaths. Thus, it seemed plausible that the virus is transmissible in public transportation to some degree [3]. As a consequence, in most countries, governments recommended avoiding unnecessary trips, especially in public mass transportation, such as disease measurement controls [5]. However, although a direct relationship has not been proven yet, public sentiment is that public transportation represents an infection risk, and use is therefore avoided.

The current COVID-19 pandemic offers a new scenery for mobility. As explained in [6], during the COVID-19 crisis a series of measures were taken to restrict travel and social activities outside the home in order to reduce the spread of the pandemic and its negative effects. These unprecedented measures have had a deep impact on the number and purpose of trips and modes of travel. Although the pandemic currently seems to be under control thanks to vaccines, and transport availability is almost back to normal, the extent of the changes in innovative mobility uses such as car sharing both while the pandemic continues and after the pandemic is over remains unclear nevertheless.

Car sharing has increased in the last ten years within both businesses and companies [7]. Car sharing has emerged in large cities as a model of shared mobility that is very convenient due to its availability, low price, and other advantages, such as sustainability (see [8] for review). In fact, previous research has pointed out that it is used as a car ownership substitution and a complement to public transportation, even in cities where there is a good public transportation system [9]. In general, consumers tend to use this type of mobility for different types of trips such as business, leisure, and other personal trips such as shopping or visiting family [10]. Car sharing is considered a social equity tool as well, ref. [11] because it can contribute to empower women by means of improving women’s possibilities for independent mobility [8]. Therefore, car sharing strategies can help governments in cities to promote the sustainable development goals (SDGs), specifically Goal 3 on gender equality, Goal 11 on sustainable cities and communities, and Goal 13 on climate action [12]. Thus, the biggest cities in the world are implementing these strategies mainly based on electric mobility in order to build more sustainable cities [7]. 

Car sharing is provided as a shared-mobility service that allows users to share a fleet of vehicles following two dominant forms: station-based and free-floating car sharing. Free-floating car sharing allows customers to temporarily use a car and leave it at the trip destination, where the next user will pick it up [9]. Companies such as Uber are not considered car sharing companies and are not included in this study, as they provide vehicles with a driver similarly to a traditional taxi, while using an application to connect drivers and users (www.uber.com, accessed on 16 December 2021).

Regarding the future of car sharing, ref. [13] noted three possible scenarios for sharing mobility: (1) a slow return to the previous normality; (2) the collapse of shared mobility; and (3) increased adoption. Nevertheless, according to the best knowledge of the author, no research has been developed in this regard. Therefore, the aim of this study, which is explorative in nature, is to investigate the usage, drivers, and barriers of car sharing usage in the ongoing pandemic era. To this end, interviews were conducted among 66 car sharing users during the COVID-19 pandemic, identifying both the drivers and barriers to car sharing usage behaviour during and after the pandemic and their effect on car sharing companies and mobility in cities. 

This research contributes to academic perspectives, business practice, and policy-making. From an academic perspective, this paper provides evidence on the dynamics of the feedback and empowerment of car sharing and contributes to shedding light on its impacts on mobility in cities; as [14] (p. 2) outlined, “the dynamics and impacts of the sharing economy are more complex than they initially seem and thus it is necessary to analyse different angles and concepts”. Moreover, this study provides direct information on mobility behaviour in times of crisis.

For business practice, this research provides fresh insight into the future of car sharing, detecting the obstacles that shared mobility will experience in the near future. Thus, it can help car sharing mobility companies to survive this crisis and complex environment, adapting their operations and services to avoid losing customer trust. Finally, for policy makers, mobility sharing should be a relevant issue for achieving more resilient cities with post-Covid futures, and can contribute to achieving the SDGs.

The rest of the paper is organised as follows. Section 2 is a review of the literature on car sharing, both before and after the lockdown due to the pandemic. Section 3 describes the methodology used in the empirical study. Section 4 presents the qualitative analysis and results. This article ends with a final section that contains an analysis of the results and conclusions based on empirical analysis, and proposes future lines of research.

## 2. Literature Review

### 2.1. Car Sharing Usage before COVID-19

We are living in changing times and mobility patterns are experiencing change. One of these new patterns of mobility is car sharing. Car sharing has added new travel choices and influenced travel mobility in cities [5].

Car sharing emerged in the last decade as a mobility system that meets the problem of transport supply in poorly served areas, particularly at night and for multiple destination trips, because it is more comfortable, flexible, and direct. Before the current COVID-19 pandemic, car sharing mainly covered journeys in the evening and on the weekend for leisure [15]. Silvestri et al. [10] reported that car sharing is suitable for business trips inside the city and other personal and social trips such as shopping and visiting friends and family. Thus, the main car sharing trip purposes reported before COVID-19 were daily short trips [15], shopping [16], holidays [15], friends and family visits, and other leisure activities [16]. In particular, this mode of transport has been found to be very suitable for moving around the city centre in cities with restrictions due to environmental issues, such as London, Rome, or Madrid [6].

Motivations for car sharing are multiple, complex, and heterogeneous. It is used for convenience and cost savings that are relevant for all socioeconomic profiles [8]. Beck and Hensher [17] summarised the benefits reported by previous research in financial, utility, environmental, and hedonic benefits. In fact, car sharing is promoting a new kind of materialism where goods are utilised more and made accessible to those who do not have ownership [8]. Regarding sustainability, several consequences have been reported. Among the most important is a reduction in CO_2_ emissions [17]. Moreover, shared vehicles can replace private vehicles or postpone the decision to purchase a car [10]. In fact, there is evidence of a reduction in car ownership in different countries (e.g., North America [7], Germany [18], Sweden [16], and Italy [15]). In consequence, there is a reduction in pollution levels and citizens’ health has improved. Thus, car sharing can contribute to the liveability of cities.

On the other hand, car sharing is a consequence of a new materialism where people prefer to have access to goods when they need them instead of buying and accumulating them [14]. Women perceive an improvement in their mobility choices from car sharing because women’s daily activities now entail multiple destinations and short trips such as shopping, children’s mobility, or family care activities [19] and it is not always easy to buy and maintain a second car [8]. Thus, car sharing contributes to both environmental sustainability and to social sustainability, empowering women [20].

### 2.2. Car Sharing during COVID-19 and Post-COVID-19

The impact of the pandemic on individual mobility depends on how policies deal with such disruptive events [2]. Silvestri et al. [10] assert that the coronavirus pandemic will change the frequency and mode of mobility even after COVID-19 is controlled. Changes in mobility are happening worldwide (e.g., [21] in Australia, and [21] in China). The distance individuals move from their home during the day has been dramatically reduced in city centres, residential areas, and small cities [15]. Nevertheless, in both developed and developing countries this is informed by a lack of adequate housing, stay at home measures [10], and the impossibility of providing totally safe transport and mobility services in times of epidemic crisis [10]. In fact, Hartl et al. [21] explain how people were being urged to avoid public transportation. Thus, people avoided travel on public transport and shared mobility because of the risks of infection [9].

The impact of the pandemic on individual mobility depends on how policies deal with such disruptive events [2]. The aforementioned researchers found an increase in car dependence. Their findings revealed a willingness to pay extra for safe, clean, comfortable, and resilient public transport. Moreover, the frequency of time spent outdoors has been reduced for many activities. In fact, people prefer shopping online and online-working [2].

Average daily kilometres travelled by car, bus, and train decreased significantly while bicycle trips increased.

Thus, people have changed their travel mode following the end of the first lockdown in cities due to the fear of getting infected [2]. Walking has been reported as the preferred way to make the shortest trips (less than one kilometre) after COVID-19, while bicycle or car are the preferred choices when the trip is longer [22]. In fact, people have substituted public transport for other motorised travel modes to support social distancing and to stay safe and healthy [10].

According to previous research [8], gender is another important variable in mobility research. Before COVID-19, more than 50% of women used public transportation, 25% used walking, and 25% used other motorised modes for shorter trips such as shopping, visiting friends, going to the gym, or to work and study, among other daily activities [2]. After COVID-19, women reduced the total time of travelling and restricted mobility to essential trips in order to reduce their exposure to the virus. Even a reduction in number of days driving per week has led to combining all the trips in a single day [21]. Thus, nearly 60% of people have avoided non-mandatory travel and stayed at home [2]. Previous research concluded that the car is perceived as the safest mode of transportation, followed by walking and riding a bicycle.

In mega cities, public transportation has dropped by 30% for primary and secondary activities [21]. The car is predominantly used by high income groups. People are more dependent on their personal mode of transportation, and the use of shared mobility dropped 35% compared to before COVID-19 [15]. People feel safer in their own vehicles compared to sitting in a shared vehicle carrying other passengers [10]. 

People that do not have their own car seek other choices for mobility other than mass public transportation. After a city is re-opened, the private vehicle clearly dominates as the most convenient choice. For this reason, people prefer ride-sharing to public transportation [21,22]. 

Previous research has found that the primary reasons for using public transport despite the pandemic are: (1) no other choice; (2) no personal vehicle; (3) convenience; and (4) it represents a cheaper option. However, 60% of people avoided travelling and preferred to stay at home; when they had to make a trip, the car was perceived as the safest mode of transportation, followed by walking and bicycle [20]. In fact, Hartl et al. [21] found that people preferred ride-sharing to using public transport. Public transport is perceived as a high COVID-19 transmitter, and others have shown very strong concerns about cleaning [21] and infection risk because public transportation does not guarantee adequate social distancing [11]. 

Nevertheless, shared mobility could be considered a hybrid between private ownership and the use of public transport. As a consequence, it could be a preferred choice over other modes of transport, being perceived as safer than other mass modes of transportation. Therefore, the research questions that will be answered here are:

RQ1: Usage of car sharing in the ongoing pandemic;

RQ2: Emerging profiles of car sharing users in the ongoing pandemic;

RQ3: Advantages, drivers and obstacles to the use of car sharing in the ongoing pandemic.

## 3. Methodology

### 3.1. Contextualisation

Shokouhyar et al. [6] suggested that mega cities such as Tokyo, Paris, or Madrid, which offer plenty of public transportation choices, are particularly vulnerable to viral transmission. Madrid was selected as a case study for several reasons. The city of Madrid is located in the centre of Spain and is the capital city of Spain. According to Wang et al. [23] (p. 250), mobility in Madrid is characterised by a dense, highly-integrated, and well-structured multimodal public transport system. It comprises 12 metro lines, 209 urban bus lines, 444 suburban bus lines, 8 suburban rail lines, and 4 tram/light rail lines. Moreover, a study [24] on the use of transportation in Madrid showed that the car is the most used form of transport. In fact, the car is used on average 17 days per month, the metro 11.7 days per month, the bus 9.5 days per month, and the train 6 days per month.

Regarding car sharing, Madrid concentrates the largest number of shared mobility operators worldwide, with 29 companies and more than 30,000 cars [25]. 

### 3.2. Method and Sample

This research focuses on the behaviour and thoughts of car sharing users after lockdown. A qualitative approach with semi-structured and open question interviews was developed. This type of question allows in-depth exploration of a topic from different perspectives [26]. This type of approach is the most suitable to acquire data when the phenomenon is new or there is very little information [6]. The interview questions are included in Appendix A.

Muller et al. [5] explained different types of factors related to using this type of mobility, namely, personal and situational factors. Personal factors are linked to a person’s characteristics. Regarding car sharing, users’ profiles are relatively young, well-educated, and from middle income families (see [8] for revision review). Situational factors include physical and social surroundings, temporal perspective, and status. In the case of shared mobility during the lockdown, in most countries activities were reduced to 2% to 5% of their regular activity and even to zero, as in the city of Madrid during the worst moments of the pandemic. 

A purposive sampling was used; this type of sampling is very useful for two reasons. First, to find relevant cases for the research with specific criteria, and second, the necessity of involving hard-to-reach populations [27]. 

In order to analyse car sharing usage after lockdown, people were selected according to the following criteria: (a) participants had to have been car sharing users before the Covid pandemic; (b) participants had to have a car sharing user profile reported by previous research [8]; (c) car sharing users had to be available and willing to participate and describe their feelings, thoughts, perceptions, and behaviour [6]. The interview questions were designed to address the research aims. Interviews were made by phone to avoid personal contact and infection, and were carried out during May to July of 2021. In order to minimise bias, the confidentiality and anonymity of the data were guaranteed. A total of 66 interviews were developed. Table 1 show the interviewees’ profiles. 

The sample was made up of 53% women and 47% men; 33% of participants were of European, Asian, and American nationality, respectively. Regarding age, 30% of the participants were below 21 years old and 100% had completed university studies or were studying for a university degree. Those characteristics agree with the profile of car sharing users provided by previous research.

### 3.3. Data Analysis

The questionnaires were introduced in Nvivo Software (QSR International, Melbourne, Australia) using thematic analysis [28]. The first coding consisted of adhesion to informed terms without restrictions, and the second round of analysis in detail in order to identify topics in a theory-driven manner. The third round consisted of identification of emerging topics [6]. Finally, the categories were combined with the topics initially identified in order to validate, refine, and expand the thematic categories. This last step allowed the identification and classification of different types of car sharing users during the pandemic.

## 4. Results

### 4.1. Car Sharing Usage during the Ongoing COVID-19 Pandemic 

First, car sharing usage was established in order to answer RQ1: Usage of car sharing in the ongoing pandemic. Most of the participants continued to use car sharing services during the pandemic as before the pandemic (74% of participants). Thus, they declared that they used car sharing to go to work or university (75%), in the city centre where there are some restrictions such as low emissions areas (75%), or urgent short trips (43%). Several of the participants’ comments confirmed this fact (see Table 2).

However, differences in the usage of car sharing can be found among men and women. Thus, 90% of women use car sharing to go shopping or carry out other family errands, while only 33% of men use this form of transport for the same activities. However, 52% of women use car sharing to go to work, while 73% of men use this transport to go to work. Trips to the city centre are similar between women and men. Nevertheless, this behaviour suggests that the number of trips is lower than before the pandemic, due to the fact that mobility continues to be restricted and complete normality has not yet returned. However, both, men and women have substituted car sharing for public transportation to go to work. 

On the other hand, 6% of the participants asserted that they use car sharing more than before the pandemic to avoid other types of public transport. Nevertheless, it is possible to identify some changes in their behaviour. Table 3 shows several of the car sharing users´ assertions about how they have increased their car sharing usage in the ongoing pandemic. Thus, participants pointed out that the possibility to increase the time using the car has been a successful factor in increasing car sharing usage. 

Finally, 20% of the participants asserted that they use car sharing services less than before the COVID-19 pandemic. This situation is due to several reasons; 10% of the participants answered that they use car sharing for unexpected or urgent trips while the pandemic is ongoing. Comments are gathered in Table 4.

Another 10% of the participants asserted that they prefer to avoid car sharing because they do not trust how the car is cleaned and disinfected. In other words, they do not feel safe and they mistrust how clean the car is (for example CSUser#60; CSUser#63) or distrust the behaviour of previous car sharing users (for example CSUser#48; CSUser#53).

Regarding the recommendation of car sharing use, 88% of participants recommend using car sharing, especially for urgent or unexpected trips. They prefer car sharing to other individual use of public transportation such as taxis.

According to the current usage of car sharing services, it is possible to identify new users’ profiles. Specifically, four different car sharing users’ profiles are emerging; these will be explained in the next section.

### 4.2. Emerging Car Sharing Profiles in the Ongoing Pandemic

The pre-pandemic car sharing user profile consists in the payment per use of the car when needed. Individuals gain the benefits of ownership of a private vehicle without the full associated costs of insurance, tax, parking, fuel costs, and others [8].

**Figure 1:**
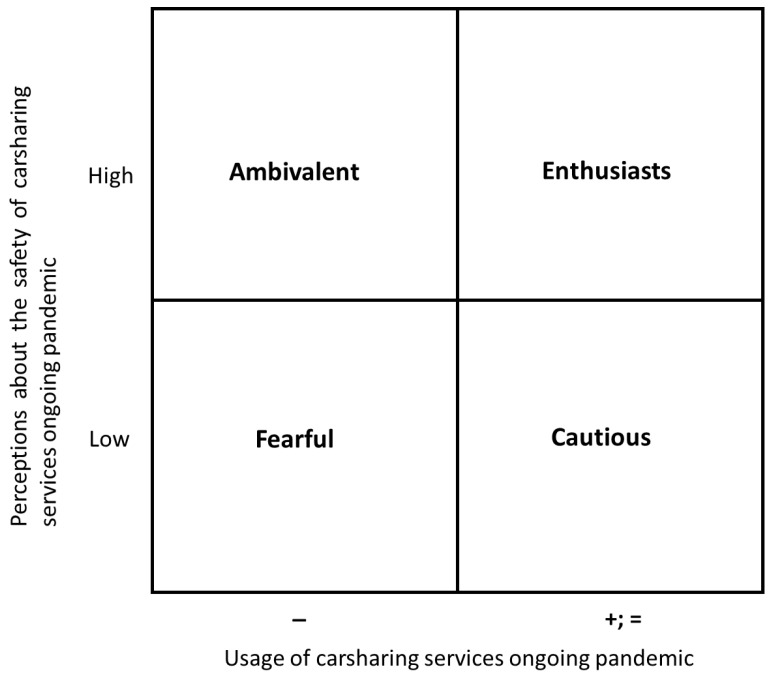
Matrix of emerging car sharing profiles in the ongoing pandemic.

However, the interviews provided new insights on car sharing profiles based on the level of car sharing usage during the ongoing pandemic and the perception of the health and safety of car sharing services. To answer RQ2: Emerging profiles of car sharing users during the ongoing pandemic, a matrix was created and four profiles were identified (see Figure 1).

The first group is labelled Enthusiasts (6% of the participants). This type of car sharing user increased their car sharing usage after the lockdown and during the pandemic. This behaviour is driven by, on the one hand, the need to make changes in daily mobility and, on the other, by the need to maintain personal distance together with the wish to avoid crowds, for example, such as those on mass public transportation. This type of user is happy to extend their car sharing use due to their trust in the car sharing mechanisms used and their cleanliness and disinfection protocols. One relevant finding is that Enthusiasts are well-informed about the measures taken by car sharing companies regarding cleanliness and disinfection. Thus, they appreciated the information campaigns on these measures and pointed out that it is one of the main reasons to use this type of service, in addition to a very good experience regarding these issues. Table 5 shows examples of their thoughts.

In addition, they take advantage of new car sharing services such as long period use, weekly, monthly, or weekends. CSUser#12 emphasises that “I have extended my usage of car sharing services. I have a car only for me. I can go to the city centre with an e-car without any restriction and I do not pay parking. It is great!” Thus, this means that in this type of car sharing services, customers can use the car without limitations as if it was their own car at a very fair price. Therefore, customers achieve high added value during the ongoing pandemic until the return of the previous situation. This new situation is profitable for car sharing providers as well, because they obtain a stable customer base and therefore a secure income to maintain their business.

The second group of car sharing users are called Cautious (46% of the participants). Cautious people are those that use car sharing in the same pattern as before the pandemic. However, they have fears about using mass public transportation, even car sharing. The main reason is that they distrust the cleanliness of the car and they are worried about the behaviour of the previous driver. Thus, participants confessed that they feel distrust regarding the cleanliness of the cars and fear that the previous user was infected or did not comply with the cleanliness and disinfection protocol. CSUser#2 “I use car sharing but always clean the car before and after its usage. I do not trust how it was cleaned before.” CSUser#31 explained “I have increased my own safety. For example, I always use a mask even though I travel alone or in spaces where it is not mandatory.”

Another problem is that sometimes users do not find any car clean nearby and it is necessary to look for another car (CSUser#4). A number of car sharing users (CSUser#27; CSUser#30; CSUser#49; CSUser#50; CSUser#59) asserted that they prefer to “use the car only for me, I distrust how the car is disinfected”.

The third group of emerging car sharing users are called Ambivalents (38% of the participants). This group use car sharing for the same short trips as before the pandemic. Nevertheless, their behaviour has changed because they do not need to make as many daily trips, in part due to the current mobility restrictions and in part due to changes in work and university study (teleworking and online classes). Thus, CSUser#19 explained “I have reduced my daily trips due to the restrictions of mobility measures, but I continue using car sharing. It is very useful for me”. Other users answered that their mobility has been reduced and they do not need any type of transportation. For example, CSUser#40 observed “I work at home and buy on the internet. I hardly leave the house. Therefore, I do not need this type of transportation, only in urgent or unexpected situations.”

Therefore, they choose different types of transport depending on the type of short trip, time of trip, or the place of trip. Thus, they mix their mobility using walking, bicycling, car sharing, or other public transportation. In addition, this group is willing to use another type of shared transport such as bicycles and scooters (CSUser#16; CSUser#51).

Finally, the last identified group is called Fearful. They represent 10% of the participants. This group distrusts car sharing services. They only use this type of transportation in very urgent situations. Moreover, they try to avoid all types of public transport and look for other ways to make essential daily short trips. They have swapped car use for walking when possible, or have bought a bicycle or a scooter, because these are cheaper than a private car and allow them to make daily short trips. In addition, users have bought their own car or family car due to their fear of sharing public transport with other people. Evidence is shown in Table 6. 

### 4.3. Advantages, Drivers and Obstacles to Car Sharing Usage during the Ongoing COVID-19 Pandemic 

Regarding RQ3: Advantages, drivers and obstacles to use of car sharing during the ongoing pandemic, participants noticed that the two main advantages of car sharing are access to individual transportation and reduction in social contact. These advantages were confirmed by 100% of participants; in fact, all participants confirmed that they preferred using car sharing to mass public transportation during the current pandemic. The most mentioned advantage was the possibility of maintaining social distance (CSUser#5; CSUser#20).

In fact, car sharing users during the ongoing pandemic asserted that they feel safe because, in addition to the new car sharing company cleanliness protocols, they can clean and disinfect the car themselves and use personal protection such as the use of plastic covers on the seats or gloves (CSUser#1). Moreover, car sharing companies have included a protection kit in the car that contains masks and alcoholic gel (CSUser#12). Another advantage is that there are more cars available than before the pandemic (CSUser#4). 

The drivers that boosted car sharing usage before the pandemic were its economic advantages, utility of service, reputation as an environmentally friendly choice, and fashion. The obstacles were mainly technological issues. Many people asserted that it is not very easy to use the app, cars were far from the user’s position, and women experienced safety problems [8].

During the pandemic, cheap prices for car sharing continued to be the main driver. Thus, price is one of most repeated drivers for car sharing usage, especially regarding the advantages provided during the pandemic +.

In the same way, utility continues to be the second driver. Car sharing services are perceived as better than owning a car because there are no hidden costs and benefits like free parking, access to low emission zones, and the possibility of driving when there are restrictions due to high levels of pollution. This utility is based on ease of use, saving time, proximity to home, and the non-necessity of buying a car.

Finally, car sharing continues to be perceived as a choice that reduces pollution and congestion. Moreover, it promotes more sustainable consumption and social equity. Nevertheless, findings have shown three types of obstacle: (1) car sharing users; (2) cars; and (3) car sharing services. First, regarding car sharing users, they are very worried about the possibility of infection. They are afraid that not all car sharing users apply the disinfection protocol in a proper way. Another fear is about using this service at night, especially for women. This obstacle has been previously identified [29]. Finally, car sharing users may find it difficult to use the mobile app [8].

Second, several obstacles are linked directly with the car. Sometimes cars need charging, and it is necessary to look for another one. Another obstacle is that certain cars are not well maintained due to mistreatment and, again, it is necessary to look for another one. Both situations are a problem when the user is in a hurry. In addition, cars are not always available at a nearby location; therefore, users have to waste time and money looking for a car (for example CSUser#9; CSUser#14; CSUser#27; CSUser#35; CSUser#52; CSUser#55). 

Finally, sometimes it is very difficult to find parking, especially at certain times, due to car sharing using regular parking and not having a specific space to park. Car sharing users complained about penalties following accidents or problems with the car (for example CSUser#12; CSUser#28; CSUser#30; CSUser#39; CSUser#55; CSUser#62; CSUser#65). 

## 5. Conclusions

A proper measure to reduce the risk of viral transmission is deep sanitisation of common surfaces on public transportation. Previous research demonstrates that viral transmission from an infected person to others is the dominant form of transmission in confined spaces such as shared mobility [5]. Although this relationship has not been proven, after the first year of the pandemic and with mobility restrictions continuing public sentiment is that public transportation represents an infection risk. This sentiment may change when the pandemic becomes more negligible. In order to protect the health of others and to achieve “normality” in daily mobility activities, this research provides relevant insights for car sharing providers, policymakers, and academia. 

First, this research provides information about car sharing mobility behaviour. This knowledge is critical for a number of reasons; it helps car sharing companies to plan their operations and budget their incomes in short and mid-term periods, helps to understand the need of citizens to achieve a “normal” situation during the ongoing pandemic, provides urban policymakers with information about what type of public transportation is preferred by their citizens, and contributes to understanding consumer behaviour in critical times.

Second, this research provides information about the valuation by customers of the new services launched to face the pandemic. Car sharing users find high value in services such as the possibility of maintaining the same car for long periods. Thus, car sharing providers have the possibility of expanding their business with new services that help to maintain social distance and avoid mass public transportation. This provides information to policymakers in order to plan public transportation.

Third, different profiles of car sharing users have been identified. According to what we know, this is the first such research that has been conducted. Therefore, car sharing providers can adapt their services to each type of car sharing profile taking into account their fears and necessities. Thus, they are able to customise their offers and widen their customer base.

Fourth, the increase in the level of information on the existence, quality, and availability of car sharing services is important and very valued by car sharing users. Before COVID-19, car sharing users were worried about finding a car near their location to save money. During the ongoing pandemic, they are worried about what the cleanliness and disinfection protocols are and value other measures such as a personal kit to disinfect the vehicle. Car sharing providers should reinforce the value of car sharing services in facing the pandemic; a direct channel to answer user questions would be appreciated. 

Finally, given that the COVID-19 pandemic may be with us for a while, it is relevant to understand mobility behaviour in cities. Car sharing providers and policymakers should work together to design the mobility services needed for citizens depending on the pandemic situation to avoid spreading the virus. Car sharing providers should assess the most effective car sharing practices in order to face the crisis and try to improve communication with users.

In future research, the advancement of car sharing adoption during the ongoing pandemic will be analysed using a quantitative approach.

## Figures and Tables

**Table 1:** Profile of car sharing participants.

User Code	Gender	Age Range	Nationality
CSUser#1	Woman	<20 years old	Asian
CSUser#2	Man	<20 years old	Asian
CSUser#3	Woman	>20 years old	Spanish
CSUser#4	Woman	<20 years old	Spanish
CSUser#5	Man	>20 years old	Spanish
CSUser#6	Woman	>20 years old	Spanish
CSUser#7	Man	>20 years old	European (not Spanish)
CSUser#8	Women	<20 years old	Spanish
CSUser#8	Woman	<20 years old	Spanish
CSUser#9	Woman	<20 years old	Spanish
CSUser#10	Man	>20 years old	Spanish
CSUser#11	Woman	>20 years old	Latin American
CSUser#12	Woman	>20 years old	Spanish
CSUser#13	Woman	>20 years old	Spanish
CSUser#14	Man	>20 years old	Spanish
CSUser#15	Man	<20 years old	European (not Spanish)
CSUser#16	Man	>20 years old	European (not Spanish)
CSUser#17	Woman	<20 years old	Spanish
CSUser#18	Man	>20 years old	Spanish
CSUser#19	Woman	>20 years old	Latin American
CSUser#20	Woman	<20 years old	Spanish
CSUser#21	Woman	>20 years old	Spanish
CSUser#22	Woman	<20 years old	Asian
CSUser#23	Man	>20 years old	Spanish
CSUser#24	Man	>20 years old	Spanish
CSUser#25	Man	<20 years old	Spanish
CSUser#26	Man	>20 years old	Spanish
CSUser#27	Woman	>20 years old	Latin American
CSUser#28	Woman	>20 years old	Latin American
CSUser#29	Woman	<20 years old	Spanish
CSUser#30	Man	<20 years old	Spanish
CSUser#31	Man	>20 years old	Spanish
CSUser#32	Man	>20 years old	Asian
CSUser#33	Woman	<20 years old	Asian
CSUser#34	Woman	>20 years old	Asian
CSUser#35	Man	<20 years old	Spanish
CSUser#36	Woman	<20 years old	Spanish
CSUser#37	Woman	>20 years old	Spanish
CSUser#38	Woman	>20 years old	Spanish
CSUser#39	Woman	>20 years old	Spanish
CSUser#40	Man	>20 years old	Spanish
CSUser#41	Man	>20 years old	Spanish
CSUser#42	Man	>20 years old	Spanish
CSUser#43	Man	<20 years old	Spanish
CSUser#44	Man	<20 years old	Latin American
CSUser#45	Woman	>20 years old	Spanish
CSUser#46	Man	>20 years old	European (not Spanish)
CSUser#47	Man	>20 years old	Spanish
CSUser#48	Woman	>20 years old	European (not Spanish)
CSUser#49	Woman	>20 years old	Spanish
CSUser#50	Woman	>20 years old	Spanish
CSUser#51	Man	>20 years old	Spanish
CSUser#52	Man	>20 years old	Spanish
CSUser#53	Woman	<20 years old	Spanish
CSUser#54	Woman	>20 years old	Spanish
CSUser#55	Woman	>20 years old	Latin American
CSUser#56	Man	>20 years old	Spanish
CSUser#57	Woman	>20 years old	Asian
CSUser#58	Man	<20 years old	Spanish
CSUser#59	Man	>20 years old	Asian
CSUser#60	Woman	>20 years old	Spanish
CSUser#61	Woman	>20 years old	Spanish
CSUser#62	Man	<20 years old	Spanish
CSUser#63	Man	>20 years old	Asian
CSUser#64	Woman	>20 years old	European (not Spanish)
CSUser#65	Woman	>20 years old	Spanish
CSUser#66	Man	<20 years old	Spanish

**Table 2:** Car sharing usage during the pandemic.

CSUser#1	“I continue to use car sharing like before the epidemic, but I pay more attention to personal protection and wash my hands and disinfect the car before and after I use it”.
CSUser#9	“After COVID my vision towards the use of this type of transport has not been altered. In other words, the trips I make are still similar to those before the pandemic. The only thing that has changed is that now I try to avoid all types of public transport as much as possible due to the high probability of contagion”.
CSUser#13	“I use car sharing to go to work since it is not safe to travel by public transport due to the crowds at rush hour”.
CSUser#18	“I use car sharing like before COVID, although now people prefer to avoid crowds and it is easy to use car sharing to avoid places with large influxes of people so as to avoid using public transport”.
CSUser#16	“I still use this type of service for the same things as before and with the same frequency”.
CSUser#19	“I use car sharing to go to work, to the doctor or daily activities like shopping”.
CSUser#23	“I make the same trips as before COVID using car sharing”.
CSUser#28	“I still use car sharing regularly because it seems a safe and clean transportation”.
CSUser#42	“I use car sharing to go to work”.
CSUser#47	“My use of car sharing has not changed. It is still ideal for short trips (going to the supermarket or work, etc...)”.
CSUser#64	“Honestly, I will continue to use car sharing in the same way that I did before pandemic”.

**Table 3:** Behaviour of participants whose car sharing usage increased.

CSUser#12	“I use this type of transport for full days.”
CSUser#15	“I use this type of transport for long periods, the car is only for me.”
CSUser#24	“I use it more because I do not take public transport and I do not have my own car.”
CSUser#25	“I use the car sharing service more because I have substituted car sharing for public transportation in my short trips.”
CSUser#28	“Car sharing is cleaner than mass public transportation.”
CSUser#66	“Car sharing is less riskier than public transportation.”

**Table 4:** Behaviour of participants that has decreased in car sharing usage.

CSUser#11; CSUser#29; CSUser#42; CSUser#54; CSUser#58.	“I only use car sharing in very urgent situations.”
CSUser#26	“I use car sharing for very sporadic trips or when I need the service immediately to get somewhere.”
CSUser#27 and CSUser#36	“I use car sharing to avoid mass public transportation when I do not have access to my family car.”

**Table 5:** Enthusiast profile behaviour.

CSUser#1	“I feel safe when I use car sharing services. The pandemic is not affecting and changing my mobility behaviour. I have always found a very clean car and, of course, I clean and disinfect the car when I finish my trip.”
CSUser#24	“I feel totally safe with car sharing. The cars are well-disinfected, and I follow the government and company recommendations such as the use of a mask and I disinfect my hands. When I finish my trip, the steering wheel, the gear box and the car door handles are disinfected. It is safer than other types of public transportation.”
CSUser#52	“I feel very satisfied with the information provided by car sharing companies. For me, the measures that have been taken are proper and sufficient. Car sharing is my preferred choice of city mobility.”
CSUser#28	“This does not describe me, but in case of doubts, inside the car there is a disinfection kit to clean the car yourself.”
CSUser#18	“I informed myself about the new car sharing disinfection and cleanliness procedure. I feel that these measures are better than the measures taken in other types of transportation such as the metro, bus or taxi.”

**Table 6:** Fearful profile behaviour.

CSUser#36	“I have swapped car sharing for walking when possible.”
CSUser#58	“I have bought a bicycle for my daily trips.”
CSUser#40	“I bought a scooter. It is cheaper than a car and it is what I need for my daily errands.”
CSUser#57	“I bought my own car.”
CSUser#20	“My family bought a family car.”
CSUser#41	“I do not see any advantage in car sharing at this moment.”
CSUser#45	“This type of transportation is unsafe.”
CSUser#54	“I do not want to use car sharing to minimise the risk of infection.”
CSUser#45; CSUser#48	“I do not use car sharing because people are very careless and do not obey the rules on cleaning and disinfection.”

## Data Availability

Not applicable.

## Appendix A

Interviews questions.

How did you use carsharing before COVID19 pandemic?

What were the advantages and disadvantages of carsharing before COVID19 pandemic?

How do you use carsharing now during ongoing COVID19 pandemic?

What are advantages and disadvantages of carsharing during ongoing COVID19 pandemic?

Do you know which carsharing measures are used to face the COVID19 pandemic?
